# Abnormal Cerebral Perfusion and Functional Connectivity in Women with Overactive Bladder

**DOI:** 10.3390/brainsci15070689

**Published:** 2025-06-27

**Authors:** Shichun Chen, Zongpai Zhang, Yakun Zhang, Kenneth Wengler, Steven Weissbart, Weiying Dai, Xiang He, Justina Tam

**Affiliations:** 1 School of Computing, State University of New York at Binghamton, Binghamton, NY 13902, USA; schen232@binghamton.edu (S.C.); zzhan145@binghamton.edu (Z.Z.); yzhan401@binghamton.edu (Y.Z.); 2 Department of Psychiatry, Icahn School of Medicine at Mount Sinai, New York, NY 10032, USA; kenneth.wengler@nyspi.columbia.edu; 3 Department of Urology, State University of New York at Stony Brook, Stony Brook, NY 11794, USA; steven.weissbart@stonybrookmedicine.edu; 4 Department of Radiology, North Shore University Hospital, Manhasset, NY 11030, USA

**Keywords:** arterial spin labeling, perfusion, functional connectivity, overactive bladder, brain–bladder control

## Abstract

**Background:** Overactive bladder (OAB) has been linked to abnormal cerebral blood flow (CBF) and functional connectivity (FC). However, findings related to CBF and FC changes in OAB remain inconsistent across the literature. **Methods:** This feasibility study employed arterial spin labeling (ASL) to investigate abnormal CBF and posterior cingulate cortex (PCC) FC in individuals with OAB, both at rest and during bladder filling. ASL images were collected from twenty-two female participants (twelve with OAB and ten healthy controls) at bladder filling volumes of 0, 50, 100, 200, 350, and 500 mL. For OAB participants, scans were obtained both at baseline and following a single-session treatment. ASL images were categorized into low-urge and high-urge conditions based on participants’ subjective urge rating during bladder filling. A flexible factorial design was implemented with three factors: subject, group (control, OAB at baseline, and OAB posttreatment), and urge state (low vs. high). **Results:** Compared to controls, OAB participants exhibited significant decreases in ΔCBF (high urge minus low urge) in the medial prefrontal cortex and increases in ΔCBF in the supramarginal region. Additionally, ΔPCC FC with the insula was reduced in OAB participants. Posttreatment, OAB participants showed increased ΔPCC FC with the postcentral and parietal (PocP), regions associated with the sensorimotor network. Notably, changes in ΔPCC-PocP FC were associated with improvements in OAB symptoms. **Conclusions:** These findings support the feasibility of using ASL to probe dysfunctional brain–bladder control mechanisms and treatment-related changes in OAB participants, highlighting the involvement of sensory processing and attention regulation in this condition.

## 1. Introduction

Overactive bladder (OAB) is a syndrome characterized by urinary urgency, usually accompanied by daytime frequency and nocturia, with or without urgency urinary incontinence (UUI) [1]. OAB affects approximately 15% of women in the US, with double the prevalence rate in women compared to men [2,3], significantly impairing overall quality of life [4]. However, the underlying pathophysiology of OAB remains unclear.

Neuroimaging studies found altered brain activities and/or functional connectivity in the insula/anterior cingulate [5,6,7], temporoparietal/supramarginal [6,8,9], prefrontal [5,10], posterior cingulate/angular gyrus [5,7,10], postcentral [10], and occipital [10] regions during bladder filling in participants with normal bladder function. In maximum bladder capacity/strong desire to void vs. empty bladder, OAB or UUI participants have been identified as having abnormal activation or functional connectivity in these specific brain regions, insula/anterior cingulate [9], temporoparietal/supramarginal [9], prefrontal [9,11], posterior cingulate/angular gyrus [9,11], and postcentral [11] regions. In a resting state, OAB participants have been shown with abnormal functional connectivity in the prefrontal, posterior cingulate, paracentral, and occipital regions [12,13]. However, the reported changes in brain function and functional connectivity associated with OAB in a resting state and during bladder filling are often inconsistent.

The magnetic resonance imaging (MRI)-based arterial spin labeling (ASL) technique, which does not require the injection of radioactive tracers or other exogenous contrast agents, has been used to measure brain perfusion [14,15] and quantify perfusion-based functional connectivity [16]. Using ASL, changes in brain perfusion from the low-urgency state (immediately after voiding) to the high-urgency state (after drinking oral fluids) in OAB participants have been reported [17]. However, no differences in bladder-filling-associated brain activity were observed in the control group. This is quite surprising because bladder distension is known to be associated with increased activation and functional connectivity within the default mode network (DMN) in healthy participants [7,8,10]. In a resting state, OAB participants have been demonstrated with abnormal FC with DMN nodes [13].

Given the reported involvement of the DMN in bladder filling and abnormalities associated with OAB in a resting state, we focused on a core region of the DMN, the posterior cingulate cortex (PCC), to investigate its abnormal functional connectivity in OAB participants during bladder filling. The primary aim of the study was to use ASL-derived brain perfusion and functional connectivity to identify brain regions that exhibit abnormal function and activation in the resting state and during bladder filling in women with OAB. We hypothesize that the high bladder filling volume is associated with increased perfusion and enhanced PCC functional connectivity with the above-mentioned brain regions in healthy controls. Conversely, in participants with OAB, we anticipate a less pronounced increase in perfusion and PCC functional connectivity during bladder filling.

## 2. Methods

### 2.1. Sample Size Justification

Using the ASL method, a previous study reported perfusion changes from low- to high-urge states in the right anterior cingulate of 4.73 ± 1.69 mL/100 g/min in OAB subjects and 1.07 ± 2.20 mL/100 g/min in controls. Our primary interest was in the interaction effect between group (OAB vs. control) and urge state (low vs. high). Accordingly, we performed a power analysis for a two-sample *t*-test comparing the perfusion changes between the two groups. Based on a mean difference of 3.66 mL/100 g/min and a pooled standard deviation of 2.77 mL/100 g/min (derived from the reported standard deviations), a sample size of 11 subjects per group was estimated to achieve 80% power at a 5% significance level. Notably, the voxel-based analysis used in our study revealed similar regions to those identified by region-based analysis in the referenced ASL study. Our study included 12 OAB subjects and 10 controls, providing sufficient power to detect the interaction effect between group and urge on perfusion.

### 2.2. Study Population and Procedures

This cross-sectional observational study was approved by the Institutional Review Board (IRB #1000210) on 18 May 2017, through the Office of Research Compliance at Stony Brook University (Stony Brook, NY, USA), and was performed in accordance with the Declaration of Helsinki. Written informed consent was obtained from all participants prior to their involvement in the study. As this study was mechanistic in nature and not a clinical trial, a clinical trial registration number is not applicable. Ten healthy female participants and twelve female OAB participants were recruited. Participants with OAB are individuals who have not responded to previous anticholinergic or beta-3 agonist therapies. All OAB participants had received optimal treatments, defined as an adequate trial of at least 4–6 weeks at a therapeutic dose, and discontinued the medications due to insufficient symptom improvement rather than adverse effects. Exclusion criteria for both groups were as follows:

- Pregnant or planning to become pregnant.
- Current urinary tract infection.
- Use of other nerve stimulation devices.
- Prior urologic use of onabotulinum toxin.
- Diagnosis of neurogenic bladder.
- Previous tibial nerve stimulation treatment.
- Inability to undergo MRI (e.g., due to metal implants or claustrophobia).
- Anatomical or medical conditions that could hinder successful placement of the PTNM electrodes.

All participants completed validated questionnaires for urinary symptoms, including the OAB-q, ICIQ-FLUTS, and UDI-6. Participants with OAB underwent a 30 min single-session PTNM treatment using the NURO^TM^ system, with a current ranging from 0.15 mA to 0.9 mA at a frequency of 20 Hz. The current level was determined based on the motor and sensory responses of each subject’s foot and toes. The OAB participants underwent the same MRI scans twice on the same day: once immediately before the treatment and once immediately after. The effects of the intervention were evaluated during the fMRI session using participant-reported bladder sensations. During bladder filling, participants pressed a button to indicate key sensory milestones: first sensation of bladder filling, first desire to void, strong desire to void, and maximum bladder capacity. These responses enabled real-time assessment of treatment-related changes in bladder sensory thresholds.

### 2.3. Study Questionnaires

The International Consultation on Incontinence Modular Questionnaire for Female Lower Urinary Tract Symptoms (ICIQ-FLUTS) and the Hospital Anxiety and Depression Scale (HADS) were administered to each participant prior to the scan. The ICIQ-FLUTS questionnaire, consisting of 12 validated items, has been extensively utilized to assess lower urinary tract symptoms in women [18]. This questionnaire contains an item measuring bother from urinary urgency—the primary symptom of OAB—and another item assessing bladder pain. The urgency bother score reflects the degree to which urgency symptoms are bothersome to the patient, while the bladder pain score represents the severity of pain experienced. Both measures are on a scale of 0 to 10, with higher scores indicating greater levels of bother. The HADS is a well-validated instrument for measuring anxiety and depression [19], with separate scores for anxiety and depression each ranging from 0 to 21. Higher scores indicate greater levels of anxiety or depression. Because participants with OAB may have elevated anxiety and depression, these scores were collected from both healthy controls and OAB participants because they could potentially confound the fMRI results.

### 2.4. MRI Scans

All participants were instructed to void their bladder first and then imaged on a 3T Prisma scanner (Siemens, Erlangen, Germany) with a 64-channel head/neck coil. A series of up to six sets of functional MRI (fMRI) images were performed at bladder filling volumes of 0, 50, 100, 200, 350, and 500 mL. Bladder filling was stopped once participants reported reaching maximum bladder capacity. Bladder filling was performed between acquisition of each set of images by infusing saline into the bladder at a rate of 50 mL/min through a urethral catheter. During bladder filling, participants were given a response button to signal: first sensation of bladder filling, first desire to void, strong desire to void, and maximum bladder capacity. fMRI images were acquired with the double echo–2D-echo-planar imaging (DE-2D-EPI) sequence, which enables simultaneous acquisition of arterial spin labeling (ASL) and blood-oxygen-level-dependent (BOLD) images with an acquisition time of 7 min 48 s for each bladder filling volume. The DE-2D-EPI parameters were: TR = 4 s, TE1 = 12 ms, TE2 = 30 ms, label duration = 1.6 s, postlabeling delay = 1.2 s, matrix size = 64 × 64, FOV = 24 × 24 cm^2^, 26 contiguous 5 mm thick slices with no gap in between. ASL images were prepared using a pseudo-continuous arterial spin labeling (PCASL) sequence [20]. High-resolution T1-weighted sagittal MPRAGE images (TR = 2.3 s, TE = 2.98 ms, TI = 900 ms, flip angle = 9°, matrix size = 240 × 256, FOV = 24 × 25.6 cm^2^, 160 contiguous 1 mm thick slices) were acquired for the purpose of registration.

### 2.5. Image Analysis

Only DE-2D-EPI ASL images were analyzed in the study. Analysis of BOLD images is in progress. ASL image analysis was performed using Statistical Parametric Mapping (SPM12, Wellcome Trust Centre for Neuroimaging, London, UK). The image registration process started by realigning all ASL control and label image time series from each subject to correct for motion, and the six affine motion parameters were subsequently regressed out. The ASL difference images were then created by performing pair-wise subtraction of label and control images. During this step, SPM also generated the mean ASL control/label image. Next, the T1-weighted image from each subject was segmented to isolate the gray matter image, which is then co-registered to the subject’s mean ASL control/label image. Using this co-registered gray matter map, all ASL difference image time series were transformed to the standard Montreal Neurological Institute (MNI) template space through the SPM normalization function.

### 2.6. Cerebral Blood Flow (CBF) Map Quantification

To mitigate the effects of physiological noises, signals from white matter and cerebrospinal fluid (CSF) were regressed out of the ASL difference image time series. The ASL difference image time series were smoothed using an 8 mm full width at half maximum (FWHM) Gaussian kernel and then converted to the absolute CBF map based on a single compartment perfusion model [21].

### 2.7. PCC Functional Connectivity (FC) Map Quantification

We found that incorporating global signal regression improved our statistical comparison in FC maps. As a result, we regressed out white matter, CSF, and global signals from the ASL difference time series. Subsequently, we smoothed the image times series using an 8 mm FWHM Gaussian kernel. A PCC FC map at each bladder volume for each participant was produced. These PCC FC maps were created by calculating the Pearson correlation coefficient between the time series from the PCC seed region of interest (ROI) and individual voxels throughout the brain. The location of the PCC seed (MNI coordinate (1, 55, 17) mm) was chosen from the previous work [22]. The PCC seed ROI was a sphere with a volume of 2 cm^3^ centered at the PCC seed. These PCC FC maps were subsequently converted to z-score maps using Fisher’s z transformation for enhanced normality. CBF and PCC FC maps were generated using MATLAB 2023b (MathWorks, Natick, MA, USA).

### 2.8. Statistical Analysis

To examine differential changes of CBF and PCC FC patterns from low- to high-urge states between control and OAB groups, while accounting for subject-related variation, we employed a flexible factorial design in SPM12 with three factors: subject, group (control, OAB/baseline, and OAB/posttreatment), and urge (low urge and high urge). The subject factor is an independent variable, while the group and the urge factors are not independent variables because OAB and OAB treatment groups involve the same participants and low- and high-urge states involve the same participants. The low-urge state was considered with bladder volumes from 0 to those before the first desire (not inclusive), while the high-urge state was considered with bladder volumes surpassing or equal to the subject’s strong desire. The flexible factorial design matrix included two types of regressors: subject (22 columns from 22 participants) and group and urge interaction (6 columns from 3 groups and 2 urge states). With the flexible factorial design, all the ASL images except those between the first desire and the strong desire were included in the statistical analysis. We tested the group and urge interaction effect to understand whether brain functional (CBF and PCC FC) changes from low- to high-urge states differ significantly between controls and OAB participants or between OAB participants at baseline and at posttreatment.

When finding any interaction effects between controls and OAB/baseline participants, we assess the functional changes from low- to high-urge states in the health control group and OAB group separately to reveal the detailed differences. In addition, we have also compared the differences in CBF and PCC FC between controls and OAB participants in each urge state. The significance maps were determined using a voxel-level *p*-value threshold of 0.005, with a cluster-level *p*-value threshold of 0.05 employed to mitigate false positives from multiple comparisons. When finding any interaction effects between OAB/baseline and OAB/posttreatment, we assess the treatment effect on brain functional changes from low- to high-urge states. In addition, we conducted post hoc regional analyses to visualize the interaction effects. The clusters identified as having significant interaction effects in the voxel-level analyses were designated as regions of interest (ROIs). We evaluated the relationship between the treatment-induced changes of OAB symptoms and changes of brain functions. Specifically, the adjusted brain CBF or PCC FC was calculated by removing subject-specific effects, i.e., subtracting the subject-specific beta value from the factorial design model. In addition, potential confounding factors such as anxiety, depression, and age were evaluated to determine their influence on the group-by-urge interaction effects on CBF and PCC FC. Linear regression models were used, with ΔCBF or ΔPCC FC (from low- to high-urge state) as the dependent variable, group (control vs. OAB) as the independent variable, and anxiety/depression and age included as covariates.

## 3. Results

The demographic data for healthy controls and OAB participants is shown in Table 1. No significant differences were observed in BMI, anxiety, and depression between the two groups. The group with OAB were older than the control group (*p* = 0.005). OAB participants reported a significantly higher urgency bother score (*p* < 0.001), pain bother score (*p* = 0.031), and bladder volumes at the first desire (*p* = 0.007) and strong desire (*p* < 0.001). Bladder volumes of the OAB participants at maximum bladder capacity were significantly larger at posttreatment.

**Figure 1:**
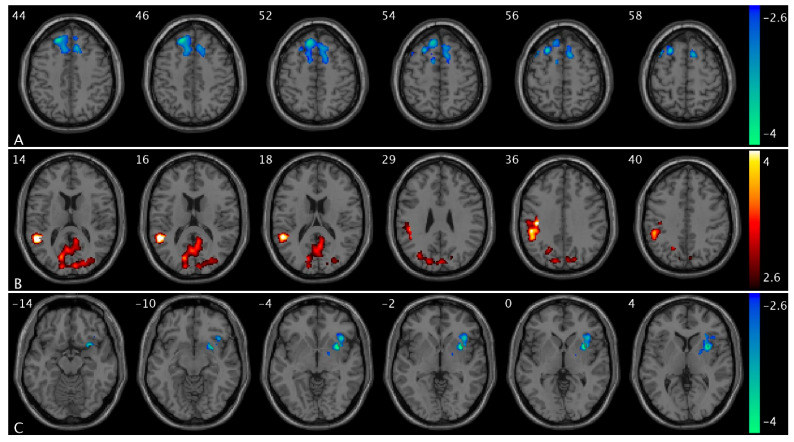
Statistical maps overlaid on T1-weighted images showing group and urge interaction effects. (**A**) Decreases in ΔCBF (from low- to high-urge state) in the superior frontal and superior medial frontal regions; (**B**) increases in ΔCBF in the cuneus, supramarginal extending to inferior parietal, middle, and superior temporal regions; (**C**) decreases in ΔPCC FC in the insula, putamen, and pallidum regions in OAB participants compared to healthy controls. The numbers in the top left corner of the MRI images indicate the z-coordinate (in mm) in the MNI space. The color bars represent t-values.

We observed significant group and urge interaction effects for CBF and PCC FC (Figure 1). Compared to healthy controls, OAB participants demonstrated significant decreases in ΔCBF (from low to high urge) in the medial prefrontal region, specifically in the superior frontal and superior medial frontal areas (Figure 1A). There were significant increases in ΔCBF (from low to high urge state) in the cuneus, supramarginal extending to inferior parietal, middle, and superior temporal regions (Figure 1B). There were significant decreases in ΔPCC FC (from low to high urge) in the insula, putamen, and pallidum regions (Figure 1C). A summary of the clusters’ statistics is reported in Table S1.

**Figure 2:**
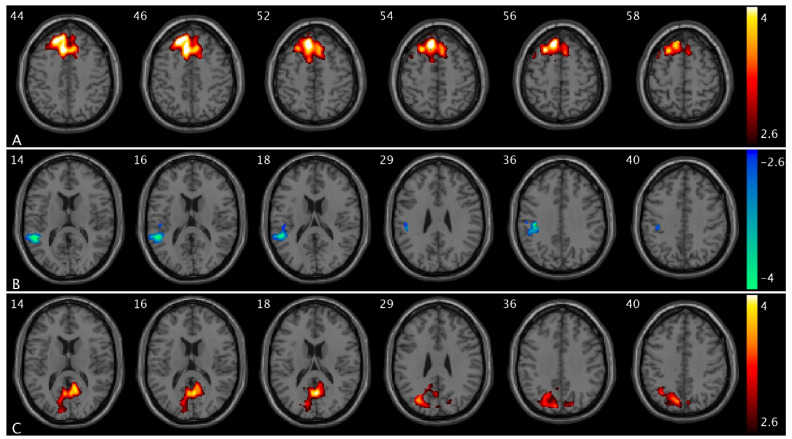
Statistical maps overlaid on T1-weighted images showing altered CBF from low- to high-urge state in healthy controls and OAB participants. (**A**) CBF increases in the superior frontal, superior medial frontal, anterior cingulate, and supplementary motor regions in healthy controls; (**B**) CBF decreases in the supramarginal, middle, and superior temporal regions in healthy controls; (**C**) CBF increases in the precuneus, cuneus, and superior occipital regions in OAB participants from low- to high-urge state. The numbers in the top left corner of the MRI images indicate the z-coordinate (in mm) in the MNI space. The color bars represent t-values.

We observed significant CBF changes from low- to high-urge states in healthy controls and OAB participants separately. Specifically, when bladder filling increases from a low- to high-urge state, we found CBF increases in the superior frontal, superior medial frontal, anterior cingulate, and supplementary motor regions in healthy controls (Figure 2A) but no CBF changes in these regions in OAB participants and CBF decreases in the supramarginal, middle, and superior temporal regions in healthy controls (Figure 2B) but no CBF changes in these regions in OAB participants; we also found no significant changes in healthy controls but CBF increases in the precuneus, cuneus, and superior occipital regions in OAB participants (Figure 2C). A summary of these clusters’ statistics is reported in Table S1.

**Figure 3:**
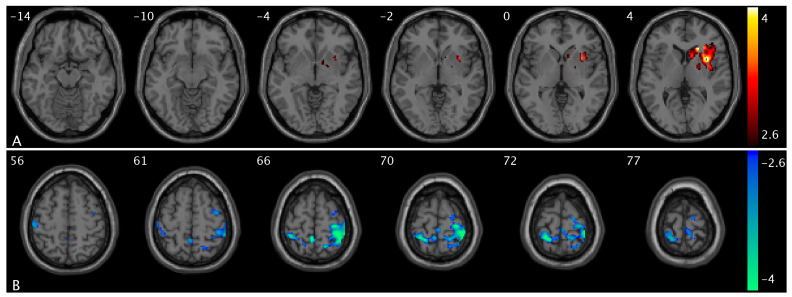
Statistical maps overlaid on T1-weighted images showing altered PCC FC from low- to high-urge state in healthy controls and OAB participants. (**A**) PCC FC increases in insula, putamen, and caudate in healthy controls (within the same slice range as in Figure 1C); (**B**) PCC FC decreases in the postcentral and superior parietal regions in healthy controls. Additional increased PCC FC regions are shown in Figure S1C. The numbers in the top left corner of the MRI images indicate the z-coordinate (in mm) in the MNI space. The color bars represent t-values.

We observed significant changes in PCC FC from low- to high-urge states in healthy controls and OAB participants separately. Specifically, when bladder filling increases from low- to high-urge states, we found PCC FC increases in the superior frontal, superior medial frontal, insula, putamen, caudate, middle temporal, and middle occipital regions in healthy controls (Figure 3A and Figure S1C) but no PCC FC changes in these regions in OAB participants; and PCC FC decreases in the postcentral and superior parietal regions (i.e., sensorimotor network area) in healthy controls (Figure 3B) but there are no PCC FC changes in these regions in OAB participants. A summary of the clusters’ statistics is reported in Table S1.

**Figure 4:**
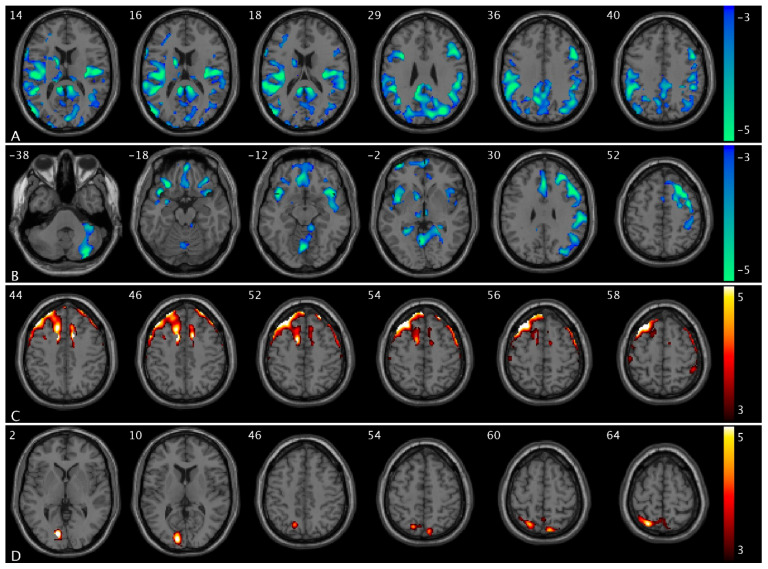
Statistical maps overlaid on T1-weighted images showing altered CBF in OAB participants compared to controls in the low- and high-urge states. (**A**) Decreased CBF the Rolandic extending to inferior frontal, precuneus, supramarginal, and angular extending to inferior parietal cortex, Heschl extending to superior and middle temporal cortex, calcarine and cuneus extending to middle and superior occipital cortex, insula, thalamus, caudate, PCC, middle cingulate cortex (within the same slice range as in Figure 1B) in OAB participants in the low-urge state; (**B**) decreased CBF in the Rolandic, rectus, precentral extending to middle, superior, inferior, and orbital frontal cortex, angular/supramarginal, Heschl extending to superior temporal cortex, vermis extending to cerebellum, insula, thalamus, caudate, pallidum, amygdala, and anterior cingulate cortex (ACC) regions in OAB participants in the high-urge state; (**C**) increased CBF in the supplementary motor extending to superior frontal and middle frontal cortex (within the same slice range as in Figure 1A) in OAB participants in the low-urge state; and (**D**) increased CBF in the inferior occipital regions in OAB participants in the high-urge state compared to healthy controls. Additional decreased CBF and increased CBF regions in OAB participants in the low-urge state are shown in Figures S1A and S1B, respectively. The numbers in the top left corner of the MRI images indicate the z-coordinate (in mm) in the MNI space. The color bars represent t-values.

We observed altered CBF in OAB participants compared to healthy controls in different urge states. Specifically, compared to controls, OAB participants exhibited decreased CBF in the Rolandic extending to inferior frontal and orbitofrontal cortex, precuneus, supramarginal, and angular extending to inferior parietal cortex, Heschl extending to superior and middle temporal cortex, calcarine and cuneus extending to middle and superior occipital cortex, vermis, insula, thalamus, caudate, PCC, middle cingulate cortex, hippocampus, and amygdala regions in the low-urge state (Figure 4A and Figure S1A) and in the Rolandic, rectus, precentral extending to middle, superior, inferior, and orbital frontal cortex, angular/supramarginal, Heschl extending to superior temporal cortex, vermis extending to cerebellum, insula, thalamus, caudate, pallidum, amygdala, and anterior cingulate cortex (ACC) regions in the high-urge state (Figure 4B) and increased CBF in the supplementary motor extending to superior frontal and middle frontal cortex, fusiform extending to inferior temporal cortex, and ACC regions in the low-urge state (Figure 4C and Figure S1B) and in the inferior occipital regions in the high-urge state (Figure 4D). A summary of the clusters’ statistics is reported in Table S1.

**Figure 5:**
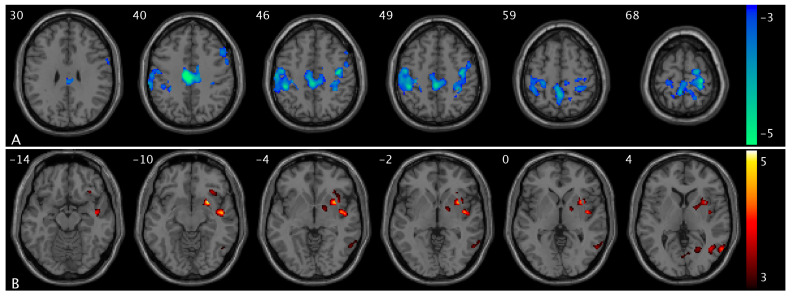
Statistical maps overlaid on T1-weighted images showing altered PCC FC in OAB participants compared to controls at different urge states. (**A**) Decreased PCC FC with the precentral, postcentral, and paracentral extending to inferior parietal cortex and middle cingulate regions and (**B**) increased PCC FC with the calcarine, insula, putamen, and pallidum regions in OAB participants compared to healthy controls in the low-urge state. The numbers in the top left corner of the MRI images indicate the z-coordinate (in mm) in the MNI space. The color bars represent t-values.

We observed altered PCC FC in OAB participants compared to controls in different urge states. Specifically, OAB participants exhibited decreased PCC FC with the precentral, postcentral, and paracentral extending to inferior parietal cortex and middle cingulate regions (Figure 5A) and increased PCC FC with the calcarine, insula, putamen, and pallidum regions (Figure 5B) in the low-urge state. A summary of the clusters’ statistics is reported in Table S1.

**Figure 6:**
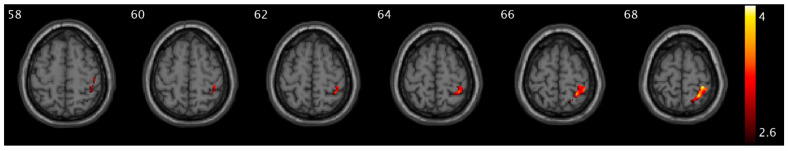
Statistical maps overlaid on T1-weighted images showing treatment effect. Increases in ΔPCC FC (from low- to high-urge state) in the postcentral and parietal regions in OAB/posttreatment compared to OAB/baseline. The numbers in the top left corner of the MRI images indicate the z-coordinate (in mm) in the MNI space. The color bar represents t-values.

**Figure 7:**
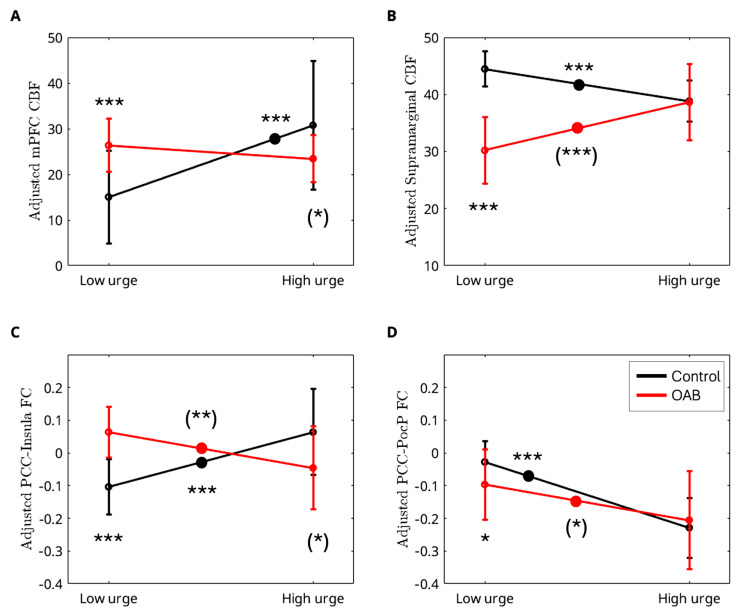
Post hoc interaction effects between group and urge states. As urge states increased from low to high: (**A**) the control group exhibited a significant increase in CBF in the mPFC region, while the OAB group showed no change; (**B**) the control group exhibited a significant decrease in CBF, while the OAB group experienced a significant increase in the supramarginal region; (**C**) the control group displayed a significant increase in PCC-insula FC, while the OAB group experienced a significant decrease in PCC-insula FC; (**D**) both the control and OAB groups exhibited a decrease in PCC-PocP FC. Bars represent standard deviations. Significance levels are indicated as follows: * 0.01 ≤ *p* < 0.05, ** 0.001 ≤ *p* < 0.01, *** *p* < 0.001, reflecting differences between controls and OABs in the low- and high-urge states and between the low- and high-urge states within each group. Large dots are added to the lines to signify significance. Significance levels in parentheses are derived solely from the post hoc regional analyses, not from the voxel-level analyses.

**Figure 8:**
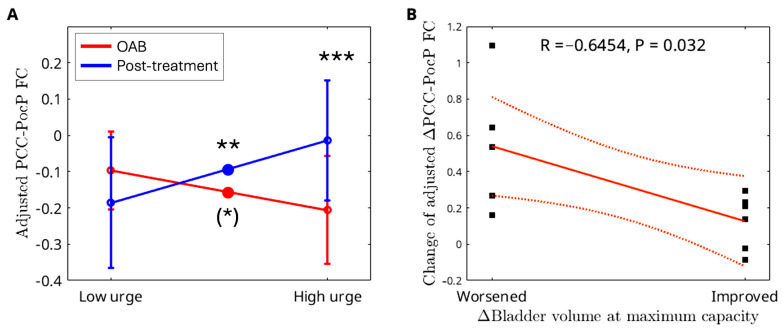
(**A**) At baseline, the OAB group experienced a decrease in PCC-PocP FC, while posttreatment, the group exhibited an increase in PCC-PocP FC. (**B**) Relationship between change of symptom (bladder volume at maximum capacity) and change of ΔPCC-PocP FC from low- to high-urge state after treatment in OAB participants. ‘Worsened’ indicates a decrease in bladder volume at maximum capacity after treatment, whereas ‘Improved’ indicates an increase in bladder volume at maximum capacity after treatment. Bars represent standard deviations. Significance levels are indicated as follows: * 0.01 ≤ *p* < 0.05, ** 0.001 ≤ *p* < 0.01, *** *p* < 0.001, reflecting differences between baseline and posttreatment of OABs in the low- and high-urge states and between the low- and high-urge states within each group. Large dots are added to the lines to signify significance. Significance levels in parentheses are derived solely from the post hoc regional analyses, not from the voxel-level analyses. Dash lines indicate the 95% confidence intervals.

Compared to OAB participants at baseline, those at posttreatment demonstrated a significant increase in ΔPCC FC (from low to high urge) in the postcentral and parietal (PocP) regions (Figure 6). A summary of the cluster statistics is reported in Table S1. Post hoc regional analyses confirmed significant interactions between group and urge states. As urge states increased from low to high, the control group showed a significant increase in CBF in the mPFC region, whereas the OAB group exhibited no change (Figure 7A). In the supramarginal region, the control group displayed a significant decrease in CBF with rising urge states, while the OAB group displayed a significant increase (Figure 7B). The control group demonstrated a significant increase in PCC-insula FC as urge states rose, while the OAB group showed a significant increase in PCC-insula FC (Figure 7C). ΔCBF values in the mPFC from low- to high-urge states were significantly correlated with anxiety scores (*p* = 0.0043), while ΔFC scores between the PCC and insula were significantly correlated with depression scores (*p* = 0.0059). However, no significant associations were found between ΔCBF or ΔFC and age (*p* > 0.05). Notably, the group-by-urge interaction effects remained unchanged after adjusting for age and anxiety/depression. Furthermore, we showed changes in PCC-PocP FC with increasing urge levels for the control and OAB groups because of the interaction between treatment and urge states. Both the control and OAB groups exhibited significant decreases in PCC-PocP FC (Figure 7D) as urge states increased. At baseline, the OAB group exhibited a significant decrease in PCC-PocP FC, but posttreatment, the group showed a significant increase in PCC-PocP FC as urge states intensified (Figure 8A). Since bladder volumes at maximum capacity were significantly larger posttreatment, we consider bladder volume a treatment-related symptom measure for OAB. We found that the OAB participants with improved symptoms (increased bladder volumes posttreatment) experienced smaller increases in ΔPCC-PocP FC (from low- and high-urge states) (Figure 8B).

## 4. Discussion

We identified significant activation in the superior medial frontal area and stronger PCC FC with the area in healthy participants when the desire to void was strong versus a low-filled bladder. Although the activation/stronger FC area is in the medial portion of the prefrontal cortex, it is not in the default mode network. Instead, it is part of the frontoparietal network. Therefore, the finding is consistent with the frequently reported lateral location of the prefrontal cortex (in the frontoparietal network) [23,24,25,26]. We have added to the literature that the medial portion of the prefrontal cortex can be activated instead of deactivated in the phase of urine storage. The heightened desire to void corresponds with activation of the superior medial frontal area, implying a potential link between the area and monitoring of interoceptive stimulation as well as perception of bladder sensation [26]. This function is in line with the role of the frontoparietal network in maintaining and manipulating information in working memory and making decisions in goal-directed behavior [27,28].

We observed that the OAB group experienced no perfusion changes, but the control group experienced increased perfusion in the superior medial frontal area during the bladder filling. These results are consistent with the activation in the superior frontal region in the healthy control groups [11,29] during the bladder filling. However, those studies reported increased activation in this region in the UUI participants [11] and in the OAB participants (the DLPFC area in their work) [29] during the bladder filling. By contrast, the literature has also shown weaker activation [25] or deactivation [29] in the OAB participants during the bladder filling. Our findings support the latter, indicating the heterogeneity of OAB participants from different studies. When the desire to void is sensed, the superior frontal region is activated, and it can determine whether to urinate according to social appropriateness. If inappropriate, it may deactivate some default mode network regions through its inhibitory connections, in line with our observed increased PCC FC with the superior frontal region during bladder filling in healthy controls. We suggest the weaker activation in this area in OAB participants may cause decreased activation in the default mode network regions (such as PCC), leading to reduced inhibition of the voiding reflex and therefore typical OAB symptoms such as UUI.

We found that bladder filling in healthy women can cause significant increases in PCC FC with the insula. A previous study has demonstrated that bladder filling may significantly activate the insula in healthy participants, and activation increases with the degree of bladder filling and desire to void [23]. This result aligns with the increased activation of brain regions within the insula and precuneus/PCC [5] and increased FC between the SN (insula and ACC) and posterior DMN [8] from bladder distention (empty bladder to strong desire to void). Pang et al. has reported increased regional homogeneity (ReHo) values in the insula of healthy adults when the bladder was full rather than empty [7]. Our results are consistent with the role of insula in receiving salient stimuli from visceral sensation (bladder distension).

We observed that the OAB group experienced no PCC FC changes, but the control group experienced increased PCC FC with the insula area during bladder filling. These results are consistent with the lack of changes in FC with bladder filling in the key brain regions, including insula, ACC, and middle frontal regions in the UUI participants [11]. Considering the key node of the insula in the salience network and its mediating role between the default mode network and the frontoparietal network, we postulate that the smaller change in functional connectivity between PCC and insula in OAB participants may be partially related to less change in functional connectivity between PCC and the superior medial frontal region during bladder filling, which led to the reduced inhibition of the voiding reflex and typical OAB symptoms as stated in the prior discussion.

Our findings in healthy participants differ from some existing studies. Prior studies have identified activation of the superior temporal/supramarginal region [6,7,9,10] and an increased nodal efficiency in the sensorimotor network (SMN, bilateral postcentral gyrus) [10] during the high-urge volumes in healthy women. In our study, the superior temporal/supramarginal region was deactivated from low- to high-urge states; the connectivity of PCC with the sensorimotor network was decreased in the high-urge state compared to the low-urge state. However, this is consistent with the deactivation in the temporoparietal junction (TPJ) and premotor cortex for the infuse phase during the high-urge volumes in controls [9]. The superior temporal/supramarginal region is a component of the ventral attention network, detecting unexpected stimuli and triggering attentional shifts [30,31]. When the bladder was filled above strong desire to void volumes, healthy controls experience deactivation in the ventral attention network and reduced FC between PCC and the sensorimotor network, indicating successfully suppressed attention and a contracted detrusor muscle/urinary sphincter. Nevertheless, differential responses in OAB participants indicate that they have abnormal attention to bladder stimuli and a relaxed detrusor muscle/urinary sphincter.

OAB participants were observed to have increased occipital activation, while healthy controls were observed with no activation in the area during the bladder filling. This is in line with the positive association between insula–occipital functional connectivity with a higher number of urgency episodes in OAB participants in a resting state [32]. The cuneus/occipital region is a part of the visual network, which processes information about object locations and adjusts visual controls of skilled movement [33]. Increased activation in this region of OAB participants may indicate a visual perception of the risk of urinary leakage in the high-urge state with strong sensation.

Decreased PCC FC with SMN was observed in OAB participants relative to controls in the low-urge state in our study. This agrees with the decreased resting state functional connectivity (rsFC) within SMN [11,13] and between SMN-aDMN [13] (anterior DMN). Interestingly, Nardos et al. also reported that DMN(AG)-SMN rsFC can be used to classify an individual as having UUI or not [11]. Meanwhile, we also found decreased perfusion in the superior temporal/supramarginal, middle occipital/cuneus, angular, and PCC regions in OAB participants regardless of the urge states (low urge or high urge) compared to healthy controls. These results are in good agreement with decreased FC in the ventral attention network and dorsal visual network [13] in the OAB group and reduced PCC FC with the DMN [34] in the urologic chronic pain syndrome group. Our results suggest decreased functional interactions of OAB participants between the DMN, SMN, ventral attention network, and dorsal visual network in a resting state, which are in the sensory processing circuit to guide the action of the bladder.

Increased PCC FC with insula (a key region of the salience network) and putamen/pallidum (basal ganglia network) was found in OAB participants compared to healthy controls in the low-urge state. A previous study reported increased dACC-caudate/thalamus FC in a resting state and increased insula–primary visual cortex FC in the low-urge state in women with urgency urinary incontinence (UUI), indirectly supporting our results of the increased FC with insula and basal ganglia area in OAB participants [35]. However, compared to healthy controls, OAB participants had reduced perfusion in the insula, putamen, and thalamus areas regardless of the filling state. We also assessed if the reduced perfusion values in these regions are affected by global signal regression, but the reduced perfusion values are still observed in OAB participants. Our results are consistent with the hypothesized brain–bladder control models [36,37]. Specifically, the bladder dilation signal is uploaded to the thalamus via PAG, then transmitted to the insula for interceptive awareness of bladder sensation. The frontoparietal network is activated through interactions with the default mode network and the basal ganglia network to support behavioral decision making. Increased functional interaction between the default mode network, salience network, and basal ganglia network in OAB participants may compensate for the reduced activity (perfusion) in those regions.

After treatment, we observed a significant increase in ΔPCC-PocC FC (from low- to high-urge state). In contrast, we found that a smaller change in ΔPCC-PocC FC (from low- to high-urge state) after treatment is associated with better outcomes. This association is somewhat surprising. Notably, both OAB/baseline (Figure 7A) and healthy controls (Figure 3B) exhibited reduced PCC-PocC FC from low- to high-urge states, thus, a decrease or small increase in ΔPCC-PocC FC (from low- to high-urge state) can bring ΔPCC-PocC FC of OAB/treatment close to those of controls, indicating an effective treatment. However, further studies are needed to determine whether ΔPCC-PocC FC can serve as a marker for treatment effects.

Our study has several limitations. First, we had a relatively small sample size. Our sophisticated model provided sufficient power to detect different brain neural responses from low- to high-urge states in OAB participants compared to healthy controls. Second, our study lacked an age-matched control group, as the OAB participants were significantly older. To address this, we included age as a covariate in the voxel-based model to mitigate the age-related effect. Additionally, we conducted region-based analyses to assess whether functional changes from low- to high-urge states were associated with age. No significant correlations were found. However, if older age is associated with subtle changes in response to bladder filling, these effects may not have been fully accounted for. Third, our study exclusively included female participants. Since bladder outlet obstruction due to prostate enlargement is a common cause of voiding symptoms in men, the underlying mechanism may differ between genders. Therefore, our findings may not be generalized to abnormal brain–bladder control mechanisms in males. Fourth, the PCASL images were acquired using 2D multi-slice acquisition. Recent background-suppressed 3D acquisitions have improved temporal SNR [20,38] and potential sensitivity to OAB differences in perfusion and functional connectivity. In addition, single postlabeling delay ASL was used in the image acquisition, which could lead to inaccuracies in absolute CBF because of variability of arterial transit time. Fifth, fMRI images were acquired at nominal bladder filling volumes of 0, 50, 100, 200, 350, and 500 mL. However, due to continuous urine production by the subjects, the actual bladder volumes at each time point often deviate from the intended targets. The average individual produces 50 mL of urine per hour, suggesting that variability in bladder volumes should be minimal. Importantly, fMRI images were categorized into low-urge and high-urge states based on subjects’ self-reported sensations, making precise bladder volumes less critical to the aims of this study.

## 5. Conclusions

PCASL MRI is feasible to identify abnormality in perfusion and PCC functional connectivity in OAB participants as they transit from a low-urge to high-urge state. In healthy women, this transition is marked by increased perfusion and stronger PCC functional connectivity with the medial prefrontal region, as well as enhanced PCC-insula functional connectivity. Concurrently, decreased perfusion is observed in the supramarginal and temporal regions. In contrast, these responses were absent in women with OAB. The heightened urge to void was associated with activation in the medial prefrontal area, suggesting a possible role for this area in interoceptive monitoring and bladder sensation perception. Following treatment, OAB participants exhibited significant increases in PCC-PocP (part of the sensorimotor network) connectivity from low- to high-urge states, which were associated with changes in OAB symptoms after treatment. Collectively, these findings suggest that PCASL MRI can detect both dysfunction and treatment-related neuroplasticity in brain–bladder control pathways, highlighting the involvement of sensory processing and attention regulation in OAB pathophysiology.

## Figures and Tables

**Table 1:** Demographic table of participants with overactive bladder and controls.

	Control (*n* = 10)	OAB (*n* = 12)	OAB Treatment (*n* = 12)	*p*-Value Control vs. OAB	*p*-Value OAB Pre vs. Post
Age (years)	28.91 ± 11.95	52.87 ± 21.30	52.87 ± 21.30 ^^^	0.0049	N/A ^⊥^
BMI (kg/m^2^)	26.36 ± 4.95	30.17 ± 5.37	30.17 ± 5.37 ^^^	0.10	N/A
Urgency bother score (0–10)	0.10 ± 0.32	7.50 ± 2.32	N/A *	<0.001	N/A
Bladder pain bother score (0–10)	0.00 ± 0.00	2.83 ± 3.83	N/A	0.031	N/A
Anxiety score (0–21)	4.60 ± 5.17	5.17 ± 3.81	N/A	0.77	N/A
Depression score (0–21)	2.00 ± 2.62	3.33 ± 2.27	N/A	0.22	N/A
Bladder volume at first sensation (mL)	75.10 ± 107.14	16.83 ± 17.69	29.17 ± 56.04	0.077	0.51
Bladder volume at first desire (mL)	165.30 ± 131.79	46.83 ± 36.92	87.08 ± 87.40	0.0072	0.21
Bladder volume at strong desire (mL)	287.00 ± 147.88	75.00 ± 45.23	132.25 ± 125.39	<0.001	0.16
Bladder volume at maximum capacity (mL)	374.80 ± 150.14	130.50 ± 88.23	269.17 ± 192.63	<0.001	0.043

^^^ Age and BMI remained unchanged because the ASL measurements at baseline and posttreatment were taken within an hour on the same day. * N/A (in this column): not available, i.e., those scores were not measured after the single-session treatment; ^⊥^ N/A (in the last column): not applicable.

## Data Availability

Participants in this study did not give written consent for their data to be shared publicly. Therefore, due to the sensitive nature of the research, the supporting data is not available.

## Supplementary Materials

The following supporting information can be downloaded at: https://www.mdpi.com/article/10.3390/brainsci15070689/s1. This includes Table S1 and Figure S1, which are referred to in the main text of the article. Table S1: Summary of cluster-level statistics; Figure S1: (A) Decreased CBF in the Rolandic extending to inferior frontal and orbitofrontal cortex, Heschl extending to superior and middle temporal cortex, calcarine and cuneus extending to middle and superior occipital cortex, vermis, insula, thalamus, caudate, PCC, hippocampus and amygdala regions in OAB participants at the low urge state. (B) increased CBF in the superior frontal and middle frontal cortex, fusiform extending to inferior temporal cortex, and ACC regions in OAB participants at the low urge state. (C) PCC FC increases in the superior frontal, superior medial frontal, insula, putamen, caudate, middle temporal, and middle occipital regions in healthy controls.
