# Supplementary material for: Abnormal Cerebral Perfusion and Functional Connectivity in Women with Overactive Bladder
Source: Brain Sci. 2025 Jun 27;15(7):689. doi: 10.3390/brainsci15070689 (PMC12294110; doi:10.3390/brainsci15070689)
Supplement: Supplementary file 1 [file brainsci-15-00689-s001.zip › brainsci-3701354-supplementary.pdf]

Supplementary Materials

Table S1. Summary of cluster-level statistics.

Figure	N Voxels	Peak-T	Coordinate	AAL3 Label	%cluster	%label
1a. ΔCBF from low to high urge: Control > OAB	1753	3.84	-18 36 46	Frontal		
				Frontal_Sup_2_L	24.70	8.89
				Frontal_Sup_Medial_L	22.25	13.03
				Supp_Motor_Area_R	11.52	8.52
				Frontal_Sup_Medial_R	11.18	9.18
				Frontal_Sup_2_R	9.70	3.32
				Supp_Motor_Area_L	6.85	5.59
				Frontal_Mid_2_L	5.53	2.15
				Limbic		
				Cingulate_Mid_R	5.08	4.04
				ACC_sup_R	2.22	7.32
				ACC_sup_L	0.40	1.16
1b. ΔCBF from low to high urge: OAB > Control	1121	4.89	-50 -46 14	Parietal		
				Parietal_Inf_L	26.49	12.14
				SupraMarginal_L	24.62	21.97
				Postcentral_L	10.53	3.03
				Angular_L	1.34	1.28
				Temporal		
				Temporal_Mid_L	19.45	4.41
				Temporal_Sup_L	17.57	8.58
	1838	3.53	-14 -76 16	Occipital		
				Cuneus_L	20.08	24.26
				Calcarine_L	13.28	10.81
				Calcarine_R	12.24	12.09
				Occipital_Sup_L	11.97	16.11
				Cuneus_R	11.59	14.96
				Occipital_Mid_L	7.67	4.32
				Occipital_Mid_R	5.11	4.48
				Occipital_Sup_R	4.68	6.09
				Parietal		
				Precuneus_R	8.38	4.72
				Precuneus_L	3.43	1.79
				Limbic		
				Cingulate_Post_R	0.60	3.28
1c. ΔPCC FC for low to high urge: Control > OAB	727	4.76	34 8 -4	Insula		
				Insula_R	47.59	19.55
				Basal Ganglia		
				Putamen_R	37.96	25.99
				Pallidum_R	5.09	13.21
				Frontal		
				Frontal_Inf_Oper_R	4.54	2.36
				Frontal_Inf_Orb_2_R	2.61	2.17

				Olfactory_R	0.83	2.08
2a. CBF for Control: High urge > Low urge	3764	4.75	-18 36 46	Frontal		
				Frontal_Sup_2_L	27.82	21.50
				Frontal_Sup_Medial_L	21.63	27.21
				Supp_Motor_Area_L	11.64	20.40
				Frontal_Sup_Medial_R	9.91	17.48
				Frontal_Mid_2_L	8.34	6.97
				Supp_Motor_Area_R	7.39	11.73
				Frontal_Sup_2_R	4.65	3.41
				Limbic		
				Cingulate_Mid_R	4.25	7.26
				ACC_sup_R	1.89	13.32
				Cingulate_Mid_L	1.33	2.58
				ACC_sup_L	0.85	5.29
				ACC_pre_R	0.32	1.85
2b.CBF for Control: Low urge > High urge	957	4.43	-52 -44 14	Temporal		
				Temporal_Mid_L	36.05	6.98
				Temporal_Sup_L	27.69	11.54
				Temporal_Inf_L	4.49	1.34
				Parietal		
				SupraMarginal_L	8.88	6.77
				Parietal_Inf_L	7.94	3.11
				Postcentral_L	5.75	1.41
				Occipital		
				Fusiform_L	4.39	1.82
				Occipital_Inf_L	1.25	1.28
				Frontal		
				Rolandic_Oper_L	2.30	2.23
2c. CBF for OAB: High urge > Low urge	2884	4.07	4 -62 18	Parietal		
				Precuneus_L	15.12	12.36
				Precuneus_R	9.92	8.76
				Parietal_Sup_L	4.68	6.54
				Parietal_Inf_L	3.09	3.64
				Angular_L	1.14	2.81
				Occipital		
				Cuneus_L	14.53	27.55
				Occipital_Sup_L	13.80	29.14
				Occipital_Mid_L	10.23	9.04
				Calcarine_L	9.43	12.05
				Calcarine_R	8.74	13.54
				Cuneus_R	6.28	12.71
				Occipital_Sup_R	1.04	2.12
				Limbic		
				Cingulate_Post_L	0.94	5.83
				Cingulate_Post_R	0.52	4.48

3a. PCC FC for Control: High urge > Low urge	650	3.71	34 -80 22	Temporal		
				Temporal_Mid_R	47.54	7.01
				Occipital		
				Occipital_Mid_R	31.85	9.87
				Occipital_Sup_R	10.00	4.60
				Calcarine_R	7.23	2.53
	1359	4.55	38 -12 12	Insula		
				Insula_R	37.31	28.64
				Basal Ganglia		
				Putamen_R	25.39	32.49
				Caudate_R	15.75	24.85
				Pallidum_R	4.42	21.43
				Frontal		
				Frontal_Inf_Oper_R	7.14	6.93
				Frontal_Inf_Tri_R	6.40	4.04
				Rolandic_Oper_R	3.38	3.46
				Temporal		
				Heschl_R	0.22	1.20
	978	4.98	-12 50 38	Frontal		
				Frontal_Sup_2_L	34.76	6.98
				Frontal_Sup_Medial_L	30.47	9.96
				Frontal_Sup_Medial_R	24.13	11.06
				Frontal_Mid_2_L	7.26	1.58
3b.PCC FC for Control: Low urge > High urge	679	4.43	-18 -46 72	Parietal		
				Postcentral_L	57.29	9.99
				Precuneus_L	24.30	4.68
				Parietal_Sup_L	14.87	4.89
	1330	4.47	30 -26 68	Parietal		
				Postcentral_R	46.47	16.17
				Parietal_Sup_R	17.74	10.62
				Paracentral_Lobule_R	7.67	12.20
				Frontal		
				Precentral_R	17.59	6.92
				Frontal_Sup_2_R	7.29	1.89
4a. CBF at low urge: Control > OAB	23316	9.12	-48 -40 22	Parietal		
				Parietal_Inf_L	4.64	44.22
				Precuneus_R	4.49	32.10
				SupraMarginal_L	3.78	70.14
				SupraMarginal_R	3.33	39.36
				Angular_R	3.27	43.49
				Precuneus_L	3.26	21.54
				Postcentral_L	2.18	13.08
				Postcentral_R	2.07	12.61
				Parietal_Inf_R	1.73	29.96
				Angular_L	1.63	32.40

Parietal_Sup_R	0.68	7.16
Parietal_Sup_L	0.57	6.49
Insula		
Insula_L	4.46	56.03
Insula_R	1.04	13.67
Temporal		
Temporal_Sup_L	4.20	42.68
Temporal_Mid_L	3.49	16.47
Temporal_Mid_R	2.49	13.18
Temporal_Pole_Sup_L	1.57	28.48
Temporal_Sup_R	1.34	9.96
Temporal_Inf_L	1.30	9.47
Temporal_Inf_R	0.89	5.85
Heschl_L	0.51	53.33
Heschl_R	0.34	31.73
Temporal_Pole_Mid_L	0.03	1.06
Occipital		
Occipital_Mid_L	3.78	26.98
Calcarine_R	3.29	41.27
Occipital_Mid_R	2.72	30.27
Cuneus_R	2.15	35.25
Occipital_Sup_L	1.47	25.04
Occipital_Sup_R	1.37	22.65
Cuneus_L	1.30	19.86
Calcarine_L	0.85	8.81
Lingual_R	0.77	7.83
Lingual_L	0.67	7.45
Occipital_Inf_L	0.52	12.96
Frontal		
Rolandic_Oper_L	2.58	60.93
Precentral_R	2.10	14.46
Rolandic_Oper_R	2.01	35.16
Frontal_Mid_2_R	1.78	8.52
Frontal_Inf_Oper_R	1.36	22.73
Frontal_Inf_Tri_R	1.24	13.39
Frontal_Sup_2_R	1.16	5.29
Frontal_Inf_Oper_L	1.12	25.24
Precentral_L	0.86	5.67
Frontal_Inf_Tri_L	0.82	7.55
OFCpost_L	0.57	23.46
Frontal_Mid_2_L	0.33	1.73
Frontal_Inf_Orb_2_L	0.15	4.42
OFCant_L	0.06	3.16
Limbic		
Cingulate_Mid_R	1.77	18.75
Cingulate_Post_L	1.48	74.73

				Cingulate_Mid_L	1.23	14.73
				Cingulate_Post_R	1.03	71.64
				Hippocampus_L	0.73	18.24
				Amygdala_L	0.64	67.73
				Hippocampus_R	0.43	10.57
				ParaHippocampal_L	0.18	4.19
				Basal Ganglia		
				Caudate_L	0.73	21.24
				Thal_PuM_R	0.48	62.36
				Thal_PuM_L	0.36	45.95
				Putamen_L	0.21	4.80
				Thal_MDm_L	0.12	23.93
				Thal_VA_L	0.10	27.38
				Thal_VL_L	0.09	7.55
				Thal_MDm_R	0.06	11.02
				Thal_PuA_L	0.06	37.14
				Thal_MDl_L	0.04	23.81
				Thal_LP_L	0.04	36.00
				Thal_PuL_L	0.03	29.63
				Thal_PuA_R	0.03	20.59
				Thal_PuL_R	0.02	14.81
				Thal_VPL_L	0.01	1.80
				Cerebellum		
				Vermis_6	0.49	30.73
				Vermis_4_5	0.47	16.39
				Cerebelum_6_L	0.41	5.61
				Cerebelum_4_5_L	0.13	2.76
				Cerebelum_4_5_R	0.05	1.28
4b. CBF at high urge: Control > OAB	656	4.5	30 -78 30	Parietal		
				Angular_R	69.21	25.91
				Occipital		
				Occipital_Mid_R	25.00	7.82
				Occipital_Sup_R	5.79	2.69
	1673	6.07	32 -86 -38	Cerebellum		
				Cerebelum_Crus1_R	35.74	22.58
				Cerebelum_6_R	27.50	25.63
				Cerebelum_Crus2_R	22.89	18.09
				Cerebelum_8_R	5.02	3.64
				Cerebelum_4_5_R	4.78	9.29
				Cerebelum_7b_R	3.77	11.80
				Cerebellum_10_R	0.24	2.52
	1933	6.64	-26 28 -18	Insula		
				Insula_L	35.39	36.81
				Frontal		
				Rolandic_Oper_L	15.57	30.47
				OFCpost_L	8.38	28.57

			Frontal_Inf_Orb_2_L	5.69	13.51
			OFCant_L	2.22	9.71
			Frontal_Inf_Oper_L	1.03	1.93
			OFClat_L	0.52	5.08
			Temporal		
			Temporal_Pole_Sup_L	12.36	18.60
			Temporal_Sup_L	9.31	7.84
			Heschl_L	3.31	28.44
			Limbic		
			Amygdala_L	2.85	25.00
			Basal Ganglia		
			Putamen_L	0.72	1.40
1596	5.08	-38 60 -2	Frontal		
			Frontal_Med_Orb_L	27.19	60.36
			Frontal_Med_Orb_R	23.93	44.63
			Frontal_Sup_2_L	11.47	3.76
			Rectus_R	9.65	20.67
			Rectus_L	9.59	17.96
			Frontal_Mid_2_L	7.02	2.49
			Frontal_Sup_Medial_L	2.13	1.14
			Frontal_Sup_Medial_R	1.57	1.17
			Limbic		
			ACC_pre_R	2.94	7.25
			ACC_sub_L	2.26	21.43
			ACC_pre_L	1.19	3.03
			ACC_sub_R	0.50	6.06
7831	7.21	26 22 52	Frontal		
			Frontal_Sup_2_R	12.46	19.04
			Frontal_Mid_2_R	11.40	18.37
			Precentral_R	7.94	18.40
			Rolandic_Oper_R	5.66	33.28
			Frontal_Inf_Oper_R	4.72	26.45
			Frontal_Inf_Tri_R	3.41	12.41
			Frontal_Sup_Medial_L	2.98	7.79
			Supp_Motor_Area_R	2.53	8.35
			Frontal_Sup_Medial_R	2.32	8.53
			OFCpost_R	1.79	24.96
			Supp_Motor_Area_L	0.96	3.49
			Frontal_Inf_Orb_2_R	0.54	4.81
			OFCant_R	0.46	5.56
			OFCmed_R	0.24	3.06
			Insula		
			Insula_R	9.95	44.01
			Parietal		
			SupraMarginal_R	9.35	37.08
			Postcentral_R	4.38	8.97
			Parietal_Inf_R	1.15	6.69

			Temporal		
			Temporal_Sup_R	4.52	11.27
			Temporal_Pole_Sup_R	3.32	19.43
			Heschl_R	1.14	35.74
			Temporal_Pole_Mid_R	0.52	3.45
			Limbic		
			Cingulate_Mid_R	2.57	9.12
			ACC_sup_L	2.35	30.41
			Cingulate_Mid_L	1.16	4.69
			ACC_sup_R	0.82	12.01
			ParaHippocampal_R	0.41	2.83
			Amygdala_R	0.22	6.85
			ACC_pre_L	0.09	1.12
			Basal Ganglia		
			Putamen_R	0.15	1.13
3073	5.88	-2 -70 -12	Cerebellum		
			Vermis_4_5	11.00	50.83
			Vermis_6	5.63	46.63
			Cerebelum_4_5_R	4.88	17.42
			Cerebelum_6_L	3.77	6.85
			Cerebelum_4_5_L	2.67	7.29
			Cerebelum_3_R	2.08	30.92
			Vermis_3	0.29	3.95
			Occipital		
			Lingual_R	9.63	12.87
			Calcarine_R	8.43	13.92
			Lingual_L	4.52	6.63
			Calcarine_L	0.91	1.24
			Basal Ganglia		
			Caudate_L	4.78	18.26
			Thal_PuM_L	3.32	55.14
			Thal_MDm_R	3.29	79.53
			Thal_PuM_R	3.29	56.74
			Thal_MDm_L	3.12	82.05
			Pallidum_L	1.59	16.72
			Putamen_L	1.59	4.90
			Thal_VL_L	1.37	15.85
			Thal_MDl_R	1.14	97.22
			Thal_MDl_L	1.11	80.95
			Thal_IL_L	0.91	54.90
			Thal_VL_R	0.55	6.56
			Thal_PuA_L	0.52	45.71
			Thal_PuA_R	0.42	38.24
			Thal_AV_R	0.36	44.00
			Thal_IL_R	0.33	19.61
			Thal_VA_L	0.33	11.90
			Thal_LP_L	0.20	24.00

				Thal_VPL_L	0.16	2.99
				Thal_VA_R	0.10	3.85
				Thal_VPL_R	0.10	1.88
				Thal_LP_R	0.07	7.14
				Thal_AV_L	0.03	5.26
				Limbic		
				Hippocampus_R	3.84	12.47
				Hippocampus_L	2.57	8.48
				Cingulate_Post_L	1.95	12.96
				Cingulate_Post_R	1.33	12.24
				ParaHippocampal_R	0.98	2.65
				Vent_Str_L	0.78	14.72
				ParaHippocampal_L	0.62	1.94
				Parietal		
				Precuneus_R	2.41	2.27
				Precuneus_L	1.99	1.73
4c. CBF at low urge: OAB > Control	1739	6.08	56 4 -38	Temporal		
				Temporal_Inf_R	48.48	23.70
				Temporal_Mid_R	11.56	4.56
				Temporal_Pole_Mid_R	6.38	9.35
				Occipital		
				Fusiform_R	22.83	15.77
				Limbic		
				Hippocampus_R	5.81	10.68
				ParaHippocampal_R	4.95	7.60
	1133	6.92	14 42 24	Limbic		
				Cingulate_Mid_R	24.98	12.85
				ACC_pre_R	10.77	18.83
				ACC_sup_R	6.80	14.45
				Frontal		
				Frontal_Sup_2_R	21.62	4.78
				Frontal_Sup_Medial_R	18.89	10.03
				Supp_Motor_Area_R	16.68	7.97
	7095	6.85	-22 24 64	Frontal		
				Frontal_Sup_2_L	24.78	36.10
				Frontal_Mid_2_L	17.35	27.31
				Frontal_Sup_Medial_L	8.09	19.18
				Supp_Motor_Area_L	6.47	21.38
				Precentral_L	6.13	12.34
				Frontal_Mid_2_R	4.95	7.22
				Precentral_R	2.92	6.12
				Frontal_Sup_2_R	2.58	3.57
				Supp_Motor_Area_R	1.99	5.95
				Frontal_Sup_Medial_R	0.75	2.48
				Parietal		
				Postcentral_L	6.19	11.28

				Postcentral_R	4.19	7.77
				Parietal_Sup_R	2.89	9.23
				Parietal_Sup_L	2.75	9.44
				Paracentral_Lobule_L	2.09	10.97
				Paracentral_Lobule_R	1.55	13.16
				Precuneus_L	1.54	3.09
				Precuneus_R	0.90	1.96
				Limbic		
				Cingulate_Mid_L	1.01	3.71
				ACC_sup_L	0.52	6.12
				ACC_pre_L	0.16	1.75
4d. CBF at high urge: OAB > Control	1497	5.26	-24 -64 64	Parietal		
				Parietal_Sup_L	28.59	20.73
				Precuneus_L	24.58	10.43
				Precuneus_R	23.38	10.72
				Parietal_Sup_R	11.76	7.92
				Postcentral_R	2.61	1.02
				Paracentral_Lobule_R	2.40	4.31
				Occipital		
				Occipital_Sup_L	3.74	4.10
				Cuneus_L	2.34	2.30
	926	7.46	-12 -82 2	Occipital		
				Calcarine_L	27.21	11.16
				Lingual_L	15.33	6.78
				Occipital_Inf_L	14.36	14.13
				Occipital_Sup_L	13.28	9.00
				Fusiform_L	11.45	4.59
				Cuneus_L	9.61	5.85
				Occipital_Mid_L	7.99	2.27
5a. PCC FC at low urge: Control > OAB	5762	6.02	-6 -20 40	Parietal		
				Postcentral_L	18.54	27.44
				Postcentral_R	11.26	16.98
				Parietal_Inf_L	6.86	16.14
				Precuneus_L	5.80	9.47
				Paracentral_Lobule_L	4.72	20.16
				Paracentral_Lobule_R	3.87	26.67
				Precuneus_R	1.86	3.28
				SupraMarginal_L	1.72	7.88
				Parietal_Inf_R	1.41	6.02
				Parietal_Sup_R	1.21	3.15
				Parietal_Sup_L	0.62	1.74
				Frontal		
				Precentral_R	11.98	20.41
				Precentral_L	2.83	4.62
				Frontal_Sup_2_R	1.91	2.15

				Frontal_Mid_2_R	1.75	2.08
				Supp_Motor_Area_R	1.42	3.46
				Frontal_Inf_Oper_R	0.50	2.07
				Limbic		
				Cingulate_Mid_L	11.44	33.95
				Cingulate_Mid_R	9.91	25.92
	712	4.81	24 -68 10	Occipital		
				Calcarine_R	75.84	29.02
				Calcarine_L	11.38	3.59
				Occipital_Sup_R	5.62	2.83
	700	4.88	44 -68 10	Temporal		
				Temporal_Mid_R	69.00	10.95
				Temporal_Inf_R	5.29	1.04
				Occipital		
				Occipital_Mid_R	25.29	8.44
5b. PCC FC at low urge: OAB > Control	1203	4.99	28 10 -8	Basal Ganglia		
				Putamen_R	25.94	29.38
				Pallidum_R	6.32	27.14
				Insula		
				Insula_R	25.60	17.40
				Frontal		
				Rolandic_Oper_R	15.21	13.75
				Frontal_Inf_Oper_R	9.64	8.29
				Frontal_Inf_Orb_2_R	4.24	5.84
				OFCpost_R	0.91	1.96
				Temporal		
				Temporal_Sup_R	10.39	3.98
6. ΔPCC FC from low to high urge: Post-treatment > baseline	595	3.95	32 -36 68	Parietal		
				Postcentral_R	64.03	9.97
				Parietal_Sup_R	33.28	8.91

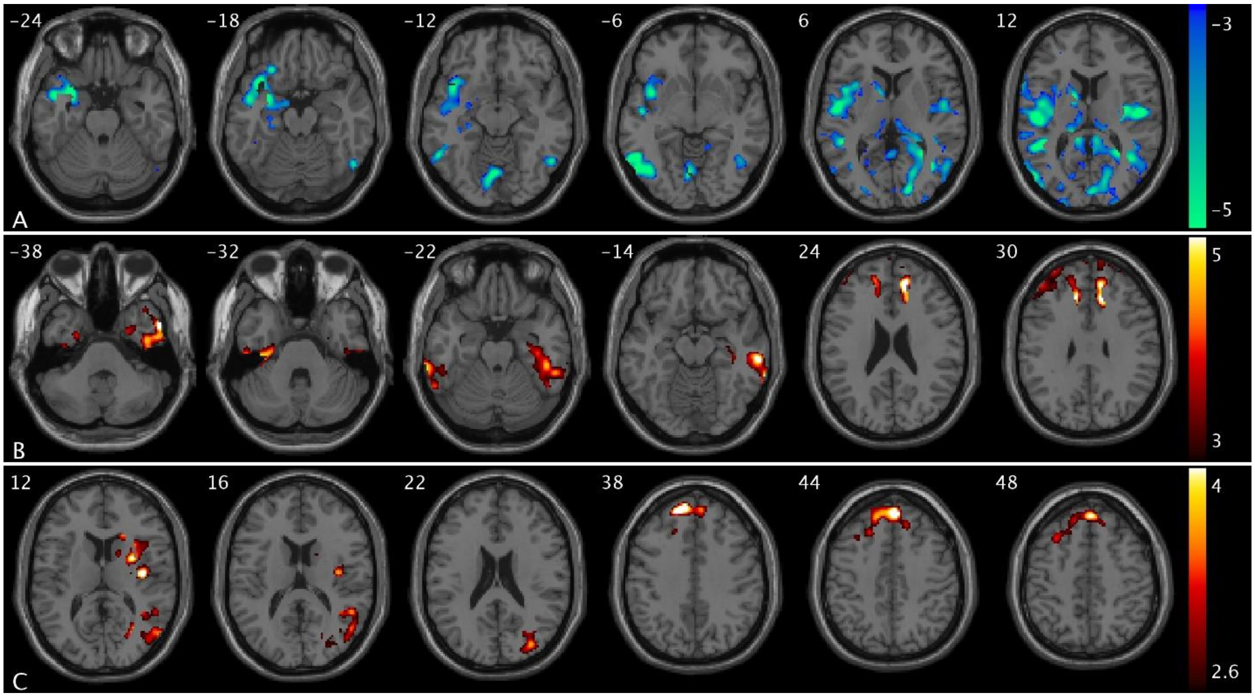

Figure S1. (A) Decreased CBF the Rolandic extending to inferior frontal and orbitofrontal cortex, Heschl extending to superior and middle temporal cortex, calcarine and cuneus extending to middle and superior occipital cortex, vermis, insula, thalamus, caudate, PCC, hippocampus and amygdala regions in OAB participants at the low urge state. (B) increased CBF in superior frontal and middle frontal cortex, fusiform extending to inferior temporal cortex, and ACC regions in OAB participants at the low urge state. (C) PCC FC increases in the superior frontal, superior medial frontal, insula, putamen, caudate, middle temporal, and middle occipital regions in healthy controls.
